# Self-reported suicidal behaviours and associated factors in the general population of Latvia (2010–2018)

**DOI:** 10.1177/00207640231174365

**Published:** 2023-05-24

**Authors:** Krista Mieze, Anda Kivite-Urtane, Daiga Grinberga, Biruta Velika, Iveta Pudule, Elmars Rancans

**Affiliations:** 1 Department of Doctoral Studies, Riga Stradins University, Latvia; 2 Institute of Public Health, Riga Stradins University, Riga, Latvia; 3 Department of Public Health and Epidemiology, Riga Stradins University, Riga, Latvia; 4 Department of Research and Health Statistics, Non-Communicable Diseases Data Analysis and Research Division, Centre for Disease Prevention and Control of Latvia, Riga, Latvia; 5 Department of Psychiatry and Narcology, Riga Stradins University, Riga, Latvia

**Keywords:** Suicidal behaviour, suicidality, suicide, Latvia

## Abstract

**Background:**

Latvia has the second highest suicide rate in the European Union – with a total population age-standardized suicide rate of 16.1 per 100,000 inhabitants.

**Aims:**

We aimed to assess the prevalence of different types of self-reported suicidal behaviours in Latvia and determine the associated sociodemographic and health-related factors.

**Methods:**

This study was based on secondary data obtained from the Health Behaviour Among Latvian Adult Population survey. A representative sample of the general population was used, aged 15 to 64 years in 2010, 2012, 2014; and 15 to 74 years in 2016 and 2018 (*n* = 16,084). Respondents were asked to report the occurrence of life weariness, death wishes, suicidal ideation, suicidal plans and suicide attempts during the previous year. We assessed socio-demographics and health-related factors associated with suicidality. We performed univariate analysis and constructed stepwise multivariate logistic regression models.

**Results:**

In 2010 to 2018, 15.6% of responders reported some type of suicidal behaviour (95% CI [15.1, 16.2]). Sociodemographic factors – including non-cohabitation status and Latvian nationality – were associated with mild (life-weariness and death wishes) and serious (suicidal ideation, plans to commit suicide, suicide attempts) types of behaviour. Older age was associated with mild suicidal behaviours, whereas lower educational levels were associated with serious suicidal behaviours. Diagnosed depression, self-reported depression, self-reported anxiety, stress, low mood, alcohol intake habits with heavy drinking episodes (less than monthly, monthly and weekly), perceived health as average or below average, disuse of primary health services were associated with mild and serious types of suicidal behaviour. Current smoking status and absenteeism were associated with mild suicidal behaviour types. Self-reported insomnia, having at least two somatic diagnoses, occasional smoking status, absenteeism with 11 or more days in the last year, receiving disability pension were associated with serious suicidal behaviour types. Musculoskeletal diseases exhibited preventive effects.

**Conclusions:**

Our findings indicate that certain groups of individuals might exhibit greater vulnerability to suicidality.

## Introduction

Suicide is one of the leading causes of death worldwide and a major public health concern. Globally, 1.3% of deaths in 2019 were due to suicide (World Health Organization [WHO], 2021b, p. 1). The most recent WHO data in 2019 reports that Latvia, a Baltic State, exhibits the second highest suicide rate in the European Union – with a total population age-standardized suicide rate of 16.1 per 100,000 inhabitants (WHO, 2021b, p. 24). The global age-standardized suicide rate was estimated as 9.0 per 100,00 individuals in 2019, which indicates that suicide is a relatively serious public health problem in Latvia (WHO, 2021b, p. 4). For several years, the suicide death rate in Latvia has remained almost twofold higher than the rate of deaths caused by traffic accidents and 4.5 times higher than the homicide death rate (Centre for Disease Prevention and Control [CDPC] of Latvia, 2019). Population research of mental disorders and suicidality in Latvia in the 2019 to 2020 revealed that at least some level of last-month suicidal behaviour was reported by 10.6% of respondents, while 7.1% reported current suicidal behaviour and 4.0% reported previous suicide attempts (Vinogradova et al., 2021).

Suicidal behaviour is a complex phenomenon involving numerous subtypes of suicidal acts, with a broad spectrum of outcomes (De Leo et al., 2006). Life weariness is an expression that indicates a low severity level, followed by, in order of increasing severity, death wishes, suicidal thoughts, suicide plans and attempted suicide, as highlighted several decades ago (Paykel et al., 1974; Tuvesson et al., 2018). Early signs of suicidality, such as suicidal thoughts, are among the most prevalent predictors of completed suicide (Dong et al., 2014; Fagerström et al., 2021). A previous episode of self-harm is associated with an increased risk of all-cause mortality – including natural and, in particular, unnatural causes – and carries the greatest risk for completed suicide within 12 months of a suicide attempt (Carr et al., 2017). This suggests that all types of suicidal behaviour are a global public health concern.

In Latvia, several studies have been conducted on suicidal behaviour in the past decades (Rancāns et al., 2003, 2016; Springe et al., 2016) as well as during the COVID-19 pandemic state of emergency (Vinogradova et al., 2021; Vrublevska et al., 2021). Although the suicide death rate in Latvia since 2008 is gradually decreasing, it remains high (WHO, 2021a). This indicates the relevance of representing tendencies and related factors of suicidality over the past decade. We aimed to assess the prevalence of different types of self-reported suicidal behaviours in the past 12 months and determine associated sociodemographic and health-related factors.

## Materials and methods

This study is based on secondary data from the Health Behaviour Among Latvian Adult Population survey in 2010, 2012, 2014, 2016 and 2018. The CDPC of Latvia provided the data. The combined stratified random sampling (random route method) and quota methods were used to select a representative sample from the general population (*n* = 16,123). The sample included residents aged 15 to 64 years (in 2010, 2012 and 2014) and 15 to 74 years (in 2016 and 2018). The sample was stratified by gender, age, location, region and nationality. Every second year, the survey was administered by trained interviewers in Latvian or Russian at the respondents’ residences. Professional interviewers with excellent knowledge in both languages and good communication skills were involved, receiving training sessions before conducting the fieldwork. These sessions were implemented in small groups up to 10 interviewers. The training covered the survey methodology, analysis of the research instrument (questionnaires), training for non-standard situations and reciprocal test interviews. All the interviewers demonstrated their understanding in aims and methods of the study, survey questions and quality standards required.

Computer-assisted personal interviews (CAPI) were used to conduct the data entry procedure. To ensure data quality 10% to 15% of the interviews were verified telephonically with the respondents by asking several key questions. Table 1 presents the total number of contacts with potential respondents and interviews. Interviews were not conducted for the following reasons: refusal to participate, non-compliance with the target group criteria, not present at their residence and the building being uninhabited. All adult patients provided verbal informed consent. Parents/guardians provided written informed consent for participants aged 15–17 years (Grīnberga et al., 2015, pp. 4–11; Grīnberga et al., 2017, pp. 5–9; Grīnberga et al., 2019, pp. 5–12; Pudule et al., 2011, pp. 5–8; Pudule et al., 2013, pp. 5–10).

**Table 1.** The number of conducted interviews and total instances of contact with potential respondents in years 2010 to 2018.

Year	Number of conducted and completed interviews	Number of total instances of contact with potential respondents (including conducted interviews)
2010	3,010	8,143
2012	3,004	5,625
2014	3,010	6,341
2016	3,596	7,864
2018	3,503	9,342
Total	16,123	37,315

The data were weighted by gender, age, type of location, region and nationality according to the Central Statistical Bureau data (the most recent data corresponding to the year the interview was conducted) to normalize it with the Latvian general population as depicted in Table 2 (Grīnberga et al., 2015, p. 8; Grīnberga et al., 2017, p. 7; Grīnberga et al., 2019, p. 8; Pudule et al., 2011, pp. 5–6; Pudule et al., 2013, p. 7).

**Table 2.** Sociodemographic distribution of the study sample before and after data weighting in years 2010 to 2018.

	Percentage of respondents in sample before weighting (%)	Percentage of respondents in sample after weighting (%)
Gender		
Men	45.9	48.1
Women	54.1	51.9
Age groups (years)		
15–34	37.4	37.5
35–54	37.3	39.2
55–74	25.3	23.3
Territory		
Urban	50.1	50.5
Rural	49.9	49.5
Ethnic groups		
Latvian	62.5	59.9
Non-Latvian	37.5	40.1
Region		
Rīga	26.0	26.4
Pierīga	14.3	14.7
Vidzeme	8.4	8.2
Kurzeme	10.5	10.5
Zemgale	10.0	9.9
Latgale	12.3	11.8

## Questionnaire and measures

Per the dependent variable, the respondents were requested to report the occurrence of their last-year suicidal behaviour based on questions designed by Paykel et al. (1974).

1. Have you ever felt that life was not worth living?
2. Have you ever wished you were dead – for instance, that you could go to sleep and not wake up?
3. Have you ever thought of taking your life, even if you would not really do it?
4. Have you ever reached the point where you seriously considered taking your life, or perhaps made plans how you would go about doing it?
5. Have you ever made an attempt to take your own life?

Response options for questions 1 to 4 were *‘often’, ‘sometimes’, ‘hardly ever’ and* ‘*never’.* For the purposes of data analysis, they were dichotomized as *‘yes’* and *‘no’* responses. The original set of responses was *‘yes’* or *‘no’* for the last question. The responses were divided into mild (solely life-weariness and death wishes) and serious (suicidal ideation, suicidal plans and suicide attempts) types of behaviour – like in previous studies (Rancāns et al., 2003, 2016). In further analysis, we included data from only those individuals who responded to all suicidal behaviour questions (*n* = 16,084). There were 39 missing responses, and 18 provided an affirmative response to at least one question on suicidal behaviour. This indicated the possibility of further ‘hidden cases’ and higher suicidality rates.

All the independent variables were divided into three groups according to the conceptual hierarchical framework model (Victora et al., 1997). We considered the interrelationships between the following related factors: proximal factors (diagnosed depression, self-reported depression, self-reported anxiety, stress and low mood), health related factors and variables influencing health as intermediate factors (self-reported insomnia, number of somatic diagnoses and self-reported cardiovascular, respiratory, musculoskeletal, gastrointestinal, urinary system diseases as well as diabetes mellitus, cancer, complaints of somatic pain, smoking status, alcohol intake habits, frequency of primary healthcare service use, self-reported health status, number of days absent and receipt of disability pension) and socio-demographic factors as distal factors (gender, age, habitat, cohabitation status, nationality, years of education, employment status and income level).

## Statistical analysis

We used SPSS version 24.0 (IBM SPSS Corp.) to perform data analysis. The statistical significance was set at *p* value <.05. We applied the logarithmic transformation to suicidality rates. Thereafter, we checked the prevalence time trends using linear regression. We applied descriptive statistics, crude odds ratios (OR) and multinomial logistic regression models. To evaluate the association between suicidal behaviour and independent variables, we constructed three stepwise logistic regression models using a conceptual hierarchical framework approach (Victora et al., 1997). In each of these, we made an adjustment using the independent variables (according to the hierarchical framework in each step) and survey year. We assessed all independent variables for collinearity (according to eigenvalues and condition indices), but multicollinearity was not detected.

## Ethical considerations

The Riga Stradins University Ethics Committee approved this study (approval no. 22-2/136/2021).

## Results

**Figure 1.**
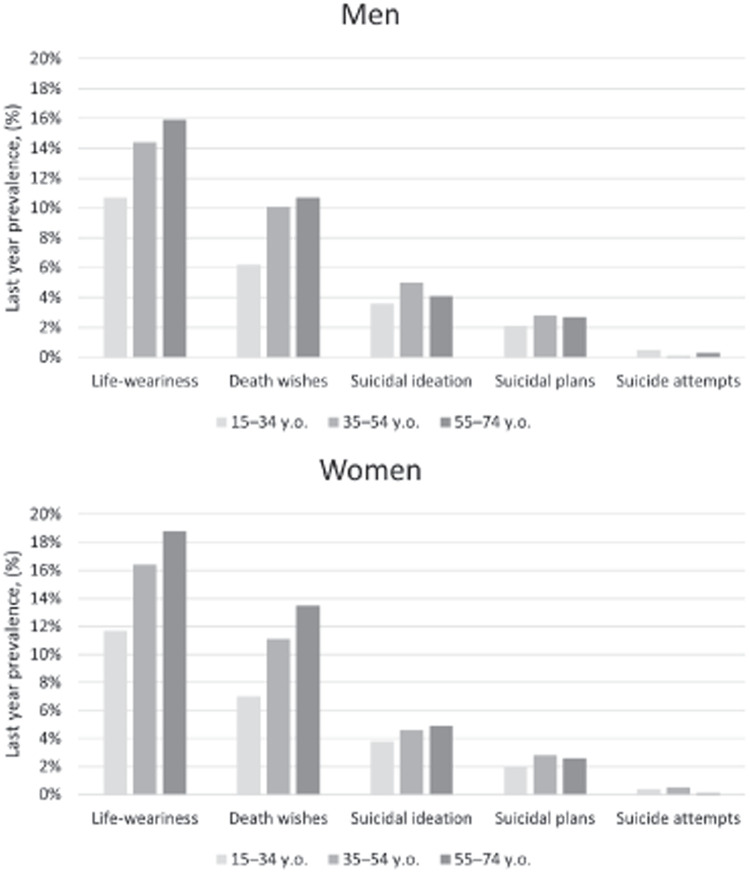
Age and gender specific last-year prevalence of self-reported suicidal behaviours in years 2010 to 2018.

Table 3 presents the prevalence of all types of self-reported suicidal behaviours in the previous year (weighted data). From 2010 to 2018, the prevalence time trends were not statistically significant: any type of suicidal behaviour was reported by 15.6% of all respondents (95% CI [15.1, 16.2]). Significant differences existed between genders (with a higher prevalence among women) in life-weariness (*p* < .001), death wishes (*p* < .001) and any type of suicidal behaviour (*p* < .001), but not in suicidal ideation, plans to commit suicide or suicide attempts. Figure 1 presents the age- and gender-specific frequencies of all types of suicidal behaviours. The prevalence of suicidal behaviours decreased with severity in both women and men across all age groups.

**Table 3.** Last year prevalence of self-reported suicidal behaviours in years 2010 to 2018 (weighted data).

Type of suicidal behaviour	2010			2012			2014			2016			2018			2010–2018		
	Male *N* (%)	Female *N* (%)	Total *N* (%)	Male *N* (%)	Fem le *N* (%)	Total *N* (%)	Male *N* (%)	Female *N* (%)	Total *N* (%)	Male *N* (%)	Female *N* (%)	Total *N* (%)	Male *N* (%)	Female *N* (%)	Total *N* (%)	Male *N* (%)	Female *N* (%)	Total *N* (%)
Life-weariness	204 (14.1%)	268 (17.6%)	472 (15.9%)	243 (16.7%)	291 (18.8%)	534 (17.8%)	126 (8.5%)	156 (10.2%)	281 (9.35)	243 (14.5%)	268 (14.0%)	511 (14.2%)	203 (12.2%)	310 (16.9%)	513 (14.6%)	1,019 (13.2%)	1,293 (15.5%)	2,311 (14.4%)
Death wishes	132 (9.1%)	180 (11.8%)	312 (10.5%)	159 (10.9%)	192 (12.4%)	351 (11.7%)	84 (5.7%)	109 (7.1%)	194 (6.4%)	153 (9.1%)	179 (9.3%)	332 (9.2%)	137 (8.2%)	207 (11.3%)	343 (9.8%)	665 (8.6%)	867 (10.4%)	1,532 (9.5%)
Suicidal ideation	74 (5.1%)	77 (5.1%)	151 (5.1%)	73 (5.0%)	66 (4.3%)	139 (4.6%)	41 (2.8%)	52 (3.4%)	93 (3.1%)	74 (4.4%)	68 (3.5%)	141 (3.9%)	66 (4.0%)	104 (5.7%)	170 (4.9%)	328 (4.2%)	367 (4.4%)	694 (4.3%)
Suicidal plans	34 (2.3%)	43 (2.8%)	77 (2.6%)	53 (3.6%)	38 (2.5%)	91 (3.0%)	16 (1.1%)	22 (1.4%)	38 (1.3%)	48 (2.9%)	44 (2.3%)	93 (2.6%)	42 (2.5%)	60 (3.3%)	102 (2.9%)	193 (2.5%)	207 (2.5%)	401 (2.5%)
Suicide attempts	5 (0.3%)	5 (0.3%)	10 (0.3%)	1 (0.1%)	5 (0.3%)	6 (0.3%)	2 (0.1%)	8 (0.5%)	10 (0.3%)	12 (0.7%)	5 (0.3%)	18 (0.5%)	5 (0.3%)	6 (0.3%)	11 (0.3%)	25 (0.3%)	29 (0.3%)	55 (0.3%)
Any type	231 (15.9%)	296 (19.4%)	527 (17.7%)	277 (19.0%)	321 (20.8%)	599 (19.9%)	140 (9.5%)	176 (11.5%)	316 (10.5%)	244 (14.5%)	272 (14.2%)	516 (14.3%)	226 (13.5%)	325 (17.7%)	551 (15.7%)	1,118 (14.5%)	1,390 (16.6%)	2,509 (15.6%)

Table 4 presents the stratified last-year prevalence of self-reported suicidal behaviour in the subgroups of independent variables (weighted data).

**Table 4.** The stratified last-year prevalence of self-reported suicidal behaviour in subgroups of the independent variables in years 2010 to 2018.

Independent variables	Types of suicidal behaviour in the past 12 months						
	None		Mild types		Serious types		*p*
	*N*	%	*N*	%	*N*	%	
Proximal factors							
Diagnosed depression in the past 12 months							
Yes	187	39.2	162	34.0	128	26.8	<.001
No	13,382	85.8	1,615	10.4	603	3.9	
Self-reported depression in the past 12 months							
Yes	2,063	55.9	1,061	28.7	576	15.4	<.001
No	11,505	92.2	718	5.8	164	1.3	
Self-reported anxiety, stress, low mood in the past 30 days							
Yes	7,005	75.7	1,581	17.1	664	7.2	<.001
No	6,568	96.1	197	2.9	68	1.0	
Intermediate factors							
Self-reported Insomnia in the past 30 days							
Yes	1,900	67.0	608	21.5	326	11.5	<.001
No	11,673	88.1	1,170	8.8	406	3.1	
Number of somatic diagnoses in the past 12 months							
None	8,605	87.8	849	8.7	333	3.4	<.001
One	2,739	82.6	412	12.4	164	4.9	
Two	1,137	78.2	213	14.7	103	7.1	
Three or more	1,088	71.5	303	19.9	131	8.6	
Cardiovascular diseases in the past 12 months							
Yes	2,313	77.1	479	16.0	207	6.9	<.001
No	11,256	86.1	1,299	9.9	524	4.0	
Respiratory diseases in the past 12 months							
Yes	602	77.2	126	16.2	52	6.7	<.001
No	12,967	84.8	1,651	10.8	679	4.4	
Musculoskeletal diseases in the past 12 months							
Yes	2,258	77.5	474	16.3	180	6.2	<.001
No	11,311	85.9	1,303	9.9	551	4.2	
Diabetes mellitus							
Yes	430	70.3	126	20.6	56	9.2	<.001
No	13,139	85.0	1,651	10.7	676	4.4	
Gastrointestinal diseases in the past 12 months							
Yes	861	73.9	208	17.9	96	8.2	<.001
No	12,708	85.2	1,569	10.5	635	4.3	
Urinary system diseases in the past 12 months							
Yes	521	72.0	132	18.2	71	9.8	<.001
No	13,048	85.0	1,646	10.7	661	4.3	
Cancer in the past 12 months							
Yes	102	65.0	34	21.7	21	13.4	<.001
No	13,467	84.6	1,744	11.0	711	4.5	
Complains of somatic pain in the past 30 days							
Yes	6,824	80.4	1,162	13.7	500	5.9	<.001
No	6,749	88.9	615	8.1	231	3.0	
Smoking status							
Current	4,067	80.7	667	13.2	308	6.1	<.001
Ex-smoker	1,796	84.9	229	10.8	91	4.3	
Occasional	479	79.2	73	12.1	53	8.8	
Never smoked	220	87.0	21	8.3	12	4.7	
Alcohol use, episodes of heavy drinking in last 12 months (six or more doses of alcohol at once)							
Less than monthly	3,280	83.5	465	11.8	184	4.7	<.001
Monthly	1,120	79.5	198	14.1	90	6.4	
Weekly	631	76.5	111	13.5	83	10.1	
Every or almost everyday	90	77.6	14	12.1	12	10.3	
Never	8,414	86.2	983	10.1	359	3.7	
Frequency of primary healthcare service use in the past 12 months							
None	3,658	86.4	396	9.4	179	4.2	<.001
1–2	5,689	86.3	656	9.9	250	3.8	
3+	4,219	80.5	724	13.8	300	5.7	
Perceived health							
Above average	8,180	91.4	582	6.5	189	2.1	<.001
Average	4,401	80.4	785	14.3	290	5.3	
Below average	983	59.7	410	6.5	253	15.4	
Number of days absent in the past 12 months							
1–10	2,911	84.3	408	11.8	133	3.9	<.001
11+	2,275	75.8	483	16.1	242	8.1	
None	8,293	87.2	874	9.2	346	3.6	
Receipt of disability pension							
Yes	755	67.5	227	20.3	136	12.2	<.001
No	12,820	85.7	1,551	10.4	596	4.0	
Distal factors							
Gender							
Men	6,614	85.5	774	10.0	345	4.5	<.001
Women	6,961	83.3	1,004	12.0	387	4.6	
Age groups (years)							
15–34	5,290	87.8	507	8.4	230	3.8	<.001
35–54	5,239	83.1	748	11.9	320	5.1	
55–74	3,046	81.2	523	13.9	181	4.8	
Territory							
Urban	6,901	84.9	852	10.5	372	4.6	.067
Rural	6,673	83.8	926	11.6	360	4.5	
Cohabitation							
Yes	7,868	86.7	884	9.7	327	3.6	<.001
No	5,706	81.5	894	12.8	405	5.8	
Nationality							
Latvian	8,115	84.2	1,082	11.2	441	4.6	.689
Non-Latvian	5,460	84.7	697	10.8	291	4.5	
Years of education							
0–9	1,341	78.7	248	14.5	116	6.8	<.001
10–13	6,917	83.6	960	11.6	397	4.8	
⩾14	5,316	87.1	570	9.3	219	3.6	
Employment status							
Employed	9,039	87.1	987	9.5	357	3.4	<.001
Unemployed	690	75.7	149	16.3	73	8.0	
Economically inactive	2,806	77.9	551	15.3	244	6.8	
Student/pupil	1,039	87.5	92	7.7	57	4.8	
Income level							
Quartile I	5,128	82.0	762	12.2	361	5.8	<.001
Quartile II	3,162	84.7	412	11.0	157	4.2	
Quartile III	1,730	87.4	185	9.3	65	3.3	
Quartile IV	2,297	87.2	243	9.2	93	3.5	

Tables 5 and 6 present the univariate and stepwise multivariate analyses results. The final model revealed that mild and serious types of self-reported suicidal behaviour were significantly associated with all proximal factors (reported diagnosed depression, self-reported depression, self-reported anxiety, stress and low mood).

**Table 5.** Factors associated with self-reported mild types of suicidal behaviour in univariate and hierarchical multivariate analysis.^a^

Mild types of suicidal behaviour												
				Model I			Model II			Model III		
	OR^b^	*p*	95% CI	aOR^c^	*p*	95% CI	aOR^d^	*p*	95% CI	aOR^e^	*p*	95% CI
Proximal factors												
Diagnosed depression in the past 12 months												
Yes	7.18	**<.001**	[5.78, 8.91]	2.08	**<.001**	[1.65, 2.62]	1.48	**.026**	[1.05, 2.09]	1.62	**.008**	[0.11, 2.33]
No	1.0			1.0			1.0			1.0		
Self-reported depression in the past 12 months												
Yes	8.24	**<.001**	[7.41, 9.16]	4.84	**<.001**	[4.32, 5.43]	3.92	**<.001**	[3.33, 4.60]	4.03	**<.001**	[3.39, 4.79]
No	1.0			1.0			1.0			1.0		
Self-reported anxiety, stress, low mood in the past 30 days												
Yes	7.54	**<.001**	[6.48, 8.78]	4.09	**<.001**	[3.48, 4.80]	3.57	**<.001**	[2.87, 4.45]	4.07	**<.001**	[3.20, 5.17]
No	1.0			1.0			1.0			1.0		
Intermediate factors												
Self-reported Insomnia in the past 30 days												
Yes	3.19	**<.001**	[2.86, 3.56]				1.28	**.007**	[1.07, 1.53]	1.21	.052	[1.00, 1.46]
No	1.0						1.0			1.0		
Number of somatic diagnoses in the past 12 months												
None	1.0						1.0					
One	1.53	**<.001**	[1.35, 1.73]				1.03	.840	[0.77, 1.37]			
Two	1.62	**<.001**	[1.62, 2.24]				0.97	.886	[0.61, 1.54]			
Three or more	2.44	**<.001**	[2.44, 3.27]				1.01	.971	[0.53, 1.94]			
Cardiovascular diseases in the past 12 months												
Yes	1.79	**<.001**	[1.60, 2.01]				1.01	.961	[0.73, 1.40]			
No	1.0						1.0					
Respiratory diseases in the past 12 months												
Yes	1.64	**<.001**	[1.35, 2.01]				1.12	.544	[0.78, 1.62]			
No	1.0						1.0					
Musculoskeletal diseases in the past 12 months												
Yes	1.82	**<.001**	[1.63, 2.05]				0.872	.348	[0.65, 1.16]			
No	1.0						1.0					
Diabetes mellitus												
Yes	2.33	**<.001**	[1.90, 2.86]				1.17	.463	[0.77, 1.79]			
No	1.0						1.0					
Gastrointestinal diseases in the past 12 months												
Yes	1.96	**<.001**	[1.67, 2.30]				1.23	.186	[0.91, 1.67]			
No	1.0						1.0					
Urinary system diseases in the past 12 months												
Yes	2.00	**<.001**	[1.64, 2.44]				1.18	.408	[0.80, 1.73]			
No	1.0						1.0					
Cancer in the past 12 months												
Yes	2.53	**<.001**	[1.71, 3.76]				0.61	.241	[0.27, 1.40]			
No	1.0						1.0					
Complains of somatic pain in the past 30 days												
Yes	1.87	**<.001**	[1.69, 2.07]				1.03	.743	[0.87, 1.22]			
No	1.0						1.0					
Smoking status												
Current	1.69	**.023**	[1.07, 2.65]				1.98	**.007**	[1.21, 3.26]	1.95	**.013**	[1.15, 3.29]
Ex-smoker	1.31	.256	[0.82, 2.09]				1.47	.146	[0.88, 2.42]	1.58	.095	[0.92, 2.71]
Occasional	1.57	.082	[0.94, 2.61]				1.76	**.048**	[1.01, 3.07]	1.41	.099	[0.91, 2.99]
Never smoked	1.0						1.0			1.0		
Alcohol use, episodes of heavy drinking in last 12 months (six or more doses of alcohol at once)												
Less than monthly	1.22	**.001**	[1.08, 1.37]				1.34	**<.001**	[1.13, 1.60]	1.45	**.001**	[1.20, 1.76]
Monthly	1.52	**<.001**	[1.29, 1.79]				1.58	**<.001**	[1.26, 1.97]	1.69	**.001**	[1.33, 2.16]
Weekly	1.51	**<.001**	[1.22, 1.87]				1.43	**.011**	[1.09, 1.88]	1.50	**.009**	[1.11, 2.04]
Every or almost everyday	1.37	.266	[0.79, 2.40]				0.79	.449	[0.43, 1.46]	0.65	.215	[0.32, 1.29]
Never	1.0						1.0			1.0		
Frequency of primary healthcare service use in the past 12 months												
None	1.0						1.0			1.0		
1–2	1.06	.358	[0.93, 1.21]				0.83	.059	[0.69, 1.01]	0.86	.136	[0.70, 1.05]
3+	1.58	**<.001**	[1.39, 1.80]				0.70	**.002**	[0.56, 0.88]	0.73	**.008**	[0.58, 0.92]
Perceived health												
Above average	1.0						1.0			1.0		
Average	2.51	**<.001**	[2.24, 2.81]				1.56	**<.001**	[1.31, 1.86]	1.46	**<.001**	[1.21, 1.76]
Below average	5.86	**<.001**	[5.08, 6.75]				2.55	**<.001**	[1.95, 3.33]	2.29	**<.001**	[1.73, 3.03]
Number of days absent in the past 12 months												
1–10	1.33	**<.001**	[1.18, 1.51]				1.21	**.050**	[1.00, 1.46]	1.37	**.003**	[1.12, 1.68]
11+	2.02	**<.001**	[1.79, 2.27]				1.28	**.015**	[1.05, 1.57]	1.41	**.002**	[1.14, 1.75]
None	1.0						1.0			1.0		
Receipt of disability pension												
Yes	2.49	**<.001**					1.32	**.047**	[1.00, 1.72]	1.17	.299	[0.87, 1.57]
No	1.0						1.0			1.0		
Distal factors												
Gender												
Men	1.0									1.0		
Women	1.23	**<.001**	[1.12, 1.36]							1.02	.795	[0.86, 1.23]
Age groups (years)												
15–34	1.0									1.0		
35–54	1.49	**<.001**	[1.32, 1.68]							1.15	.157	[0.95, 1.41]
55–74	1.79	**<.001**	[1.57, 2.04]							1.33	**.029**	[1.03, 1.71]
Territory												
Urban	1.0									1.0		
Rural	1.13	**.020**	[1.02, 1.24]							1.19	**.050**	[1.00, 1.42]
Cohabitation												
Yes	1.0									1.0		
No	1.40	**<.001**	[1.26, 1.54]							1.51	**<.001**	[1.28, 1.78]
Nationality												
Latvian	1.05	.395	[0.94, 1.16]							1.25	**.012**	[1.05, 1.48]
Non-Latvian	1.0									1.0		
Years of education												
0–9	1.72	**<.001**	[1.47, 2.02]							1.20	.204	[0.90, 1.60]
10–13	1.30	**<.001**	[1.16, 1.45]							1.07	.507	[0.88, 1.29]
⩾14	1.0									1.0		
Employment status												
Employed	1.24	.061	[0.99, 1.55]							0.84	.409	[0.56, 1.27]
Unemployed	2.44	**<.001**	[1.85, 3.22]							1.44	.130	[0.90, 2.29]
Economically inactive	2.23	**<.001**	[1.77, 2.81]							0.82	.378	[0.53, 1.28]
Student/pupil	1.0									1.0		
Income level												
Quartile I	1.41	**<.001**	[1.21, 1.64]							1.07	.593	[0.84, 1.37]
Quartile II	1.23	**.014**	[1.04, 1.46]							0.93	.579	[0.71, 1.21]
Quartile III	1.01	.904	[0.83, 1.24]							0.84	.269	[0.61, 1.15]
Quartile IV	1.0									1.0		

*Note*. OR = odds ratio; aOR = adjusted odds ratio; CI = confidence interval.

a Cells in the table are left empty due to the stepwise multivariate analysis model, according to the conceptual hierarchical framework (see Statistical analysis section in Methods for more detailed information).

b OR, Crude odds ratio.

c aOR, adjusted odds ratio in Model I, adjusted for all proximal factors (independent variables) and year of the survey.

d aOR, adjusted odds ratio in Model II, adjusted for all proximal and intermediate factors (independent variables) and year of the survey.

e aOR, adjusted odds ratio in Model III, adjusted by all proximal factors, intermediate factors (insomnia, smoking status, alcohol use, use of primary healthcare service, perceived health, absenteeism and receipt of disability pension), all distal factors and year of the survey.

Values in bold indicates a significant difference from the reference group.

**Table 6.** Factors associated with self-reported serious types of suicidal behaviour in univariate and hierarchical multivariate analysis.^a^.

Serious types of suicidal behaviour												
				Model I			Model II			Model III		
	OR^b^	*p*	95% CI	aOR^c^	*p*	95%CI	aOR^d^	*p*	95% CI	aOR^e^	*p*	95% CI
Proximal factors												
Diagnosed depression in the past 12 months												
Yes	15.24	**<.001**	[12.0, 19.36]	3.41	**<.001**	[2.65, 4.40]	2.61	**<.001**	[1.80, 3.77]	2.81	**<.001**	[1.91, 4.12]
No	1.0			1.0			1.0			1.0		
Self-reported depression in the past 12 months												
Yes	19.31	**<.001**	[16.21, 23.11]	10.94	**<.001**	[8.99, 13.32]	8.90	**<.001**	[6.78, 11.68]	9.81	**<.001**	[7.36, 13.10]
No	1.0			1.0			1.0			1.0		
Self-reported anxiety, stress, low mood in the past 30 days												
Yes	9.20	**<.001**	[7.15, 11.84]	3.18	**<.001**	[2.43, 4.18]	2.98	**<.001**	[2.05, 4.34]	3.26	**<.001**	[2.18, 4.89]
No	1.0			1.0			1.0			1.0		
Intermediate factors												
Self-reported Insomnia in the past 30 days												
Yes	4.94	**<.001**	[4.23, 5.76]				1.59	**<.001**	[1.25, 2.03]	1.48	**.002**	[1.15, 1.91]
No	1.0						1.0			1.0		
Number of somatic diagnoses in the past 12 months												
None	1.0						1.0			1.0		
One	1.55	**<.001**	[1.28, 1.88]				1.36	.126	[0.92, 2.01]	1.31	.113	[0.94, 1.84]
Two	2.34	**<.001**	[1.86, 2.95]				1.84	**.048**	[1.01, 3.35]	1.68	**.019**	[1.09, 2.59]
Three or more	3.11	**<.001**	[2.52, 3.85]				1.96	.113	[0.85, 4.53]	1.79	**.024**	[1.08, 2.96]
Cardiovascular diseases in the past 12 months												
Yes	1.93	**<.001**	[1.63, 2.28]				0.84	.410	[0.54, 1.28]			
No	1.0						1.0					
Respiratory diseases in the past 12 months												
Yes	1.66	**<.001**	[1.24, 2.22]				0.81	.411	[0.50, 1.33]			
No	1.0						1.0					
Musculoskeletal diseases in the past 12 months												
Yes	1.64	**<.001**	[1.37, 1.95]				0.599	**.009**	[0.41, 0.88]	0.67	**.021**	[0.48, 0.94]
No	1.0						1.0			1.0		
Diabetes mellitus												
Yes	2.52	**<.001**	[1.90, 3.37]				0.85	.565	[0.48, 1.49]			
No	1.0						1.0					
Gastrointestinal diseases in the past 12 months												
Yes	2.24	**<.001**	[1.79, 2.80]				1.10	.963	[0.68, 1.51]			
No	1.0						1.0					
Urinary system diseases in the past 12 months												
Yes	2.69	**<.001**	[2.07, 3.48]				1.30	.278	[0.81, 2.10]			
No	1.0						1.0					
Cancer in the past 12 months												
Yes	3.82	**<.001**	[2.37, 6.17]				0.816	.644	[0.34, 1.94]			
No	1.0						1.0					
Complains of somatic pain in the past 30 days												
Yes	2.14	**<.001**	[1.82, 2.51]				0.93	.561	[0.73, 1.19]			
No	1.0						1.0					
Smoking status												
Current	1.43	.245	[0.78, 2.61]				1.64	.150	[0.84, 3.20]	1.50	.246	[0.76, 2.98]
Ex-smoker	0.96	.887	[0.51, 1.79]				0.99	.984	[0.49, 1.99]	1.04	.918	[0.51, 2.12]
Occasional	2.08	**.028**	[1.08, 4.00]				2.49	**.015**	[1.19, 5.19]	2.26	**.034**	[1.06, 4.78]
Never smoked	1.0						1.0			1.0		
Alcohol use, episodes of heavy drinking in last 12 months (six or more doses of alcohol at once)												
Less than monthly	1.31	**.003**	[1.09, 1.58]				1.40	**.014**	[1.07, 1.84]	1.45	**.012**	[1.09, 1.93]
Monthly	1.88	**<.001**	[1.48, 2.39]				1.89	**<.001**	[1.36, 2.62]	1.93	**<.001**	[1.35, 2.74]
Weekly	3.08	**<.001**	[2.39, 3.96]				2.75	**<.001**	[1.94, 3.90]	2.87	**<.001**	[1.96, 4.22]
Every or almost everyday	3.09	**<.001**	[1.67, 5.71]				1.37	.403	[0.65, 2.88]	1.15	.725	[0.52, 2.57]
Never	1.0						1.0			1.0		
Frequency of primary healthcare service use in the past 12 months												
None	1.0						1.0			1.0		
1–2	0.90	.271	[0.74, 1.09]				0.85	.255	[0.63, 1.13]	0.83	.235	[0.61, 1.13]
3+	1.45	**<.001**	[1.20, 1.75]				0.61	**.004**	[0.44, 0.85]	0.59	**.004**	[0.41, 0.84]
Perceived health												
Above average	1.0						1.0			1.0		
Average	2.86	**<.001**	[2.37, 3.44]				1.56	**.002**	[1.18, 2.06]	1.48	**.009**	[1.00, 1.98]
Below average	11.18	**<.001**	[9.15, 13.65]				4.24	**<.001**	[2.98, 6.01]	2.75	**<.001**	[2.56, 5.47]
Number of days absent in the past 12 months												
1–10	1.09	.394	[0.89, 1.34]				1.08	.603	[0.81, 1.45]	1.14	.419	[0.83, 1.56]
11+	2.55	**<.001**	[2.15, 3.03]				1.41	**.015**	[1.07, 1.85]	1.48	**.009**	[1.10, 1.98]
None	1.0						1.0			1.0		
Receipt of disability pension												
Yes	3.88	**<.001**	[3.17, 4.74]				1.62	**.005**	[1.16, 2.28]	1.47	**.038**	[1.02, 2.12]
No	1.0						1.0			1.0		
Distal factors												
Gender												
Men	1.0									1.0		
Women	1.07	**<.001**	[0.92, 1.24]							1.05	.722	[0.81, 1.36]
Age groups (years)												
15–34	1.0									1.0		
35–54	1.41	**.003**	[1.11, 1.66]							0.86	.419	[0.59, 1.25]
55–74	1.36	**<.001**	[1.18, 1.67]							1.02	.876	[0.77, 1.36]
Territory												
Urban	1.0									1.0		
Rural	1.001	.992	[0.86, 1.16]							1.08	.559	[0.84, 1.39]
Cohabitation												
Yes	1.0									1.0		
No	1.71	**<.001**	[1.47, 1.99]							1.87	**<.001**	[1.48, 2.36]
Nationality												
Latvian	1.02	.807	[0.88, 1.19]							1.56	**<.001**	[1.22, 22.0]
Non-Latvian	1.0									1.0		
Years of education												
0–9	2.09	**<.001**	[1.66, 2.64]							1.48	**.045**	[1.01, 2.19]
10–13	1.39	**<.001**	[1.18, 1.65]							0.995	.969	[0.76, 1.31]
⩾14	1.0									1.0		
Employment status												
Employed	0.72	**.023**	[0.54, 0.96]							0.88	.663	[0.50, 1.55]
Unemployed	1.92	**<.001**	[1.34, 2.76]							1.15	.686	[0.60, 2.20]
Economically inactive	1.58	**.002**	[1.18, 2.12]							0.86	.621	[0.47, 1.58]
Student/pupil	1.0									1.0		
Income level												
Quartile I	1.74	**<.001**	[1.38, 2.20]							1.15	.473	[0.79, 1.66]
Quartile II	1.23	.126	[0.94, 1.60]							0.97	.861	[0.65, 1.44]
Quartile III	0.94	.687	[0.68, 1.29]							0.82	.418	[0.51, 1.32]
Quartile IV	1.0									1.0		

*Note*. OR = odds ratio; aOR = adjusted odds ratio; CI = confidence interval.

a Cells in the table are left empty due to the stepwise multivariate analysis model, according to the conceptual hierarchical framework (see Statistical analysis section in Methods for more detailed information).

b OR, crude odds ratio.

c aOR, adjusted odds ratio in Model I, adjusted for all proximal factors (independent variables) and year of the survey.

d aOR, adjusted odds ratio in Model II, adjusted for all proximal and intermediate factors (independent variables) and year of the survey.

e aOR, adjusted odds ratio in Model III, adjusted by all the proximal factors, intermediate factors (insomnia, number of somatic diagnoses, musculoskeletal diseases, smoking status, alcohol use, use of primary healthcare services, perceived health, absenteeism and receipt of disability pension), all distal factors and year of the survey.

Values in bold indicates a significant difference from the reference group.

Several intermediate factors remained statistically significant with mild types of suicidal behaviour in the final hierarchical model: current status of active smoking (compared to those who have never smoked), alcohol intake habits with episodes of heavy drinking in the last 12 months and using six or more doses of alcohol at once (persons who drink less than monthly, monthly and weekly compared to those who responded *‘never’*). There were higher odds for those who did not use primary healthcare services in the last year compared to those who visited three or more times. Those who perceived their health as average or below average were also associated with mild types of suicidal behaviour.

Serious types of suicidal behaviour exhibited significantly higher odds for those who: had insomnia in the past month, had more than one somatic diagnosis in the past 12 months (compared to those with none), were occasional smokers (compared to those who *’never smoked’*) and had not used primary care health services in the last year (versus those who used services three or more times). There were higher odds for serious types of suicidal behaviour for those who perceived their health as average or below average in comparison to those who responded ‘*above average*’. Absenteeism at work for 11 or more days in the past 12 months was associated with serious types of suicidal behaviour (compared to those who had none). Furthermore, the receipt of disability pension also exhibited a significant association with serious types of suicidal behaviour. Alcohol intake habits and respondents’ perceived health exhibited the same association with serious and mild types of suicidal behaviour. Musculoskeletal diseases in the past 12 months exhibited a preventive effect on serious types of suicidal behaviours. Respondents with diagnosed musculoskeletal diseases exhibited 0.67 times (or 33%) lower odds (*p* = .021) of having serious types of suicidal behaviour than those who had not been diagnosed.

Distal factors, which remained statistically significant with both mild and serious types of suicidal behaviour, were living alone (compared to those who were cohabiting) and Latvian nationality (compared with non-Latvians). Additionally, the older age (55–74 years) group, compared to the youngest age group, exhibited a significant association with mild types of suicidal behaviour but not with serious types. A lower educational level (0–9 years) was significantly associated with serious types of suicidal behaviour compared to those who had 14 years of education or more.

## Discussion

To our knowledge this is the first study in Latvia including such a large population-based representative sample of the general population, analysing varied self-reported suicidal behaviours in Latvia at the national level from a 10-year period. Our most relevant findings revealed that respondents with last year self-reported depression demonstrated 4.03- and 9.81-fold higher odds of having mild and serious types of suicidal behaviour, respectively, remaining statistically significant after adjusting for numerous socio-demographic and health related factors. The same association remained with the self-reported anxiety, stress, low mood in the past month exhibiting 4.07 and 3.26 higher odds, respectively, indicating the relevance of timely diagnosis of mental health disorders.

In this study we performed the analysis of data obtained from a period before the COVID-19 pandemic. Although the survey was also administered in 2020, including these data was impossible owing to the changes in methodology, implemented because of various pandemic-related restrictions. Two similar studies were conducted in Latvia in 2000 (Rancāns et al., 2003) and 2010 (Rancāns et al., 2016), wherein self-reported suicidal behaviour was assessed using the same questions. The first study from 2000 (Rancāns et al., 2003) used a postal survey of a stratified proportional sample of the Latvian general population aged 18 years and older (*n* = 667), which did not allow a direct comparison of these findings. The second study conducted in 2010 (Rancāns et al., 2016) was based on a representative sample of the Latvian general population aged 18 to 64 years (*n* = 2,816) using the same methodology. Our study also contained the same data as that utilized in the latter study.

Compared to the results from the 2000 study (Rancāns et al., 2003), the prevalence of different types of last-year self-reported suicidal behaviour in 2018 decreased dramatically for any type of suicidal behaviour (52.6%–15.7%), life-weariness (36.3%–14.6%), death wishes (38.7%–9.8%), suicidal ideation (12.2%–4.9%), suicidal plans (12.2%–2.9%) and suicide attempts (1.8%–0.3%). Due to different data collection methods (from postal surveys to face-to-face interviews), suicidal behaviours were possibly underreported, and hence, caution must be exercised when comparing these data. Since 2010 the prevalence of life-weariness, death-wishes, suicidal ideation, and any type of suicidal behaviour slightly decreased. The prevalence of suicide plans slightly increased, and the rate of self-reported suicide attempts remained the same. The completed suicide rate per 100,000 population indirectly confirmed these findings. The suicide rate in Latvia is gradually decreasing, with an age-standardized suicide mortality rate of 29.6 in 2000, 18.6 in 2010 and 14.3 per 100,000 in 2018 (WHO, 2021a). However, the prevalence time trends have not demonstrated statistical significance in our study.

### We found various socio-demographic factors to be significantly associated with suicidality

Our results revealed a higher prevalence of mild types and any type of suicidal behaviour among women than among men. However, the results of the final multivariate analysis model exhibited no statistical differences between genders in mild or serious types of suicidal behaviour. In the 2000 study (Rancāns et al., 2003), women reported significantly less serious types of suicidal behaviours during the last year than men. Suicide gender ratios vary across countries. The highest men-to-women suicide ratio is observed in East European countries and the former Soviet Republics; the lowest ratios are observed in Asian countries, suggesting that culturally rooted inequality determine these differences. The literature suggests that women face varied social disadvantages, and their lower suicide mortality rate indicates women’s resilience in responding to stress and crises (Chang et al., 2019). Although women face greater suicide risk factors, males exhibit an increased likelihood of transitioning from suicidal ideation to attempts (Fadoir et al., 2020). The risk of suicide attempts declines with age, with no significant interaction between suicidal ideation and age for suicide attempts or death by suicide (Rossom et al., 2017). This indirectly confirms our findings that older age is significantly associated with mild – but not serious – types of suicidal behaviour.

In our study, non-cohabitation status was related to both mild and serious suicidality. A population-based Norwegian study found that never being married or being separated, divorced or widowed was associated with an increased risk of suicide compared to being married. The risk was strongest during the first month of separation and decreased subsequently. After more than a year, it is only slightly higher than that for divorced men (Næss et al., 2021). As the final model did not show the gender as a factor significantly associated with mild and serious types of suicidality, we did not differentiate the cohabitation status in relation to suicidal behaviour in women and men. Latvian nationality in our study demonstrated 1.25- and 1.56-fold higher odds to be related with mild and serious suicidality, respectively. In 2000 Latvian nationality exhibited a significant influence on serious types of suicidal behaviour (Rancāns et al., 2003). The literature suggests that such inequalities between nations are attributable to various geopolitical processes and may also be the consequence of a certain nationality becoming a non-privileged minority (Värnik et al., 2005). The suicide risk for European women with a low education level was 1.32 times higher than those with a high educational level. In men, it was 2.5 times higher. Educational inequalities affecting suicide should be addressed in early life by targeting groups that struggle to complete their education and exhibit a higher risk of mental health vulnerabilities or disorders (Lorant et al., 2021). This is consistent with our findings, as lower educational level was associated with serious types of suicidal behaviour. Although we found that rural habitat was significantly associated with mild suicidal behaviour, another Swedish study reported high rates of attempted and completed suicides in rural and semi-rural municipalities (San Sebastián et al., 2020). Substance use, economic distress and poor access to healthcare are commonly identified explanatory factors (Mohatt et al., 2021).

### The final model in our study revealed that all the proximal factors remained statistically significant in relation to suicidality

A population-based study during the COVID-19 pandemic state of emergency in Latvia revealed that respondents with a self-reported history of clinical depression were significantly more likely to exhibit suicidal thoughts than those who did not (13.3% vs. 5.4%). Respondents with a history of at least one suicide attempt (6.13%) were more likely to meet the criteria of depression (23.13% vs. 4.61%) and distress (20.63% vs. 6.98%) and report increased suicidal thoughts during the state of emergency (27.05% vs. 4.62%) compared to those without previous suicide attempts (Vrublevska et al., 2021). Suicidal ideation varies according to the severity of depressive symptoms, and minimal symptoms of depression are uncommon (4%); reportedly, 11%, 25% and 47% individuals report mild, moderate and moderately severe to severe depression, respectively (Rossom et al., 2017). This confirms our findings that among those who exhibited diagnosed depression, mild and serious types of suicidal behaviour were present in 34.0% and 26.8% of respondents, respectively. Among those with self-reported depression, mild and serious types of suicidal behaviour were present in 28.7% and 15.4% of respondents, respectively. Those with self-reported anxiety, stress and low mood in the previous 30 days also reported mild (17.1%) and serious (7.2%) types of suicidal behaviour.

### Our study revealed that numerous health related factors exhibited significant association with suicidal behaviour

We found that insomnia was a crucial factor in relation to serious suicidality; however, some studies have suggested that insomnia exhibits no direct effect because depression significantly mediates the relationship between total sleep time and suicidal behaviour (Michaels et al., 2017). In a nationwide cross-sectional study of Latvia’s primary care population in 2015, suicidality was observed in 18.6% of patients (17.1% with low-risk suicidality). During the last 30 days, 1.9% experienced suicidal ideation, 0.5% had a suicide plan, 0.1% reported a history of suicide attempts and 4.1% reported lifetime suicide attempts – with no statistically significant differences between genders. Higher odds of current suicidality are associated with women; lower educational level; unemployment status; economically inactive employment status; being single, divorced or widowed; and living separately or in a small city (Rancans et al., 2020). In a US study, the prevalence of suicidal ideation among respondents in mental and primary health clinics was 24% and 17%, respectively (Rossom et al., 2017). Although we found a strong relationship exists between suicidality and depression, disuse of primary care services in the last 12 months was associated with suicidality. However, another study using the same sample from 2012 reported that using any health care services during the last 12 months was higher among those with depression (Vrublevska et al., 2016).

A study from Sweden reported that individuals attributing their suicide attempts to somatic distress did not differ between those with and without serious somatic illness. Two-thirds of those with serious physical illness and half without reported psychological pain as a reason for the attempt, and 23% with serious physical illness provided no explanation, suggesting that psychological processes play a greater role in the suicidality of individuals with poorer physical health (Wiktorsson et al., 2016). Among medically serious suicide attempts, 13.7% had orthopaedic diseases (Mento et al., 2015). Our findings are considerably controversial, with musculoskeletal diseases presenting as a possible preventive factor for serious suicidality. This is because of the multifactorial nature of forming suicidal phenomena. The use of health services in such a group of individuals might be higher, and musculoskeletal pain can be relieved with antidepressants.

In our findings, current smoking status significantly influenced mild types and occasional smoking on serious types of suicidal behaviour. Current smoking status is associated with elevated suicide death risk; heavy smokers also exhibit greater than twice a risk than light smokers (Evins et al., 2017). Additionally, non-daily smoking and former smoking status are related to suicidal ideation and suicide plans and attempts (Kwan et al., 2020). Alcohol consumption is a well-known risk factor at the time of suicidal action and self-harm (Hurzeler et al., 2021; Larkin et al., 2017). Our results revealed that alcohol intake habits with at least six standard doses on one occasion less than monthly, monthly or weekly was significantly associated with both types of suicidal behaviour. There was no association between any type of suicidality and daily or almost daily alcohol intake. A previous German study reported that one-third of suicide attempts were made by individuals in a state of acute alcohol intoxication, 17% of whom could be diagnosed with alcohol-use disorders. Individuals with alcohol-use disorders choose low-risk suicide methods under the influence of alcohol. Alcohol might elevate the risk by lowering natural anxiety toward self-mutilation (Boenisch et al., 2010).

Although employment status was not a relevant factor associated with suicidality, other researchers have found that employed individuals with high suicidal ideation levels exhibited 45% work productivity impairment due to absenteeism and 1.4 times greater per patient per month indirect costs (Benson et al., 2021). An increased risk of suicide attempts was observed in those who were granted a disability pension; this was four times higher for those with a disability due to mental disorders than for those with a disability due to somatic diseases (Mittendorfer-Rutz et al., 2014). We found that individuals who were granted a disability pension exhibited higher odds for serious suicidal behaviour. However, we did not differentiate the reason of the disability pension.

A core strength of this study is that it included a large population-based representative sample of the general population, analysing varied self-reported suicidal behaviours in Latvia at the national level. Furthermore, it included a broad spectrum of age groups and data from a 10-year period. Trained interviewers conducted CAPI, which eliminated possible errors in the data-entry process. However, this study’s methodological limitations should be considered. The study design – a cross-sectional survey – did not allow the evaluation of the causality of suicidality, as the survey did not contain questions on other mental disorders, lifetime or family history of suicidal behaviours. The structure of self-reported questionnaires can cause overestimation or underestimation of suicidal behaviours or other measures. Recall bias shapes potential inaccuracies in reporting primary healthcare utilisation and physician-diagnosed somatic illnesses. Additionally, responders and non-responders may exhibit different characteristics (Korkeila et al., 2001).

## Conclusion

This is the first study in Latvia to report the prevalence of different types of self-reported suicidal behaviour in the last year, representing data from the entire decade. From 2010 to 2018, the prevalence time trends have not demonstrated statistical significance in all types of suicidal behaviour. Our findings indicated that certain groups of individuals might exhibit greater vulnerability to suicidal behaviour. This could assist health providers in improving targeted suicide prevention measures at the national level. Different levels of associated factors, proximal, intermediate and distal should be considered in relation to suicidality. We recommend that the main strategies must include the improvement of screening for suicide risk, especially in the primary care setting.
